# Sickle cell knowledge, premarital screening and marital decisions among local government workers in Ile-Ife, Nigeria

**DOI:** 10.4102/phcfm.v1i1.22

**Published:** 2009-06-23

**Authors:** Emmanuel A. Abioye-Kuteyi, Olanrewaju Oyegbade, Ibrahim Bello, Chiddude Osakwe

**Affiliations:** 1 Department of Community Health, Obafemi Awolowo University, Nigeria; 2 Department of General Medical Practice, Obafemi Awolowo University Teaching Hospitals’ Complex, Nigeria

**Keywords:** premarital screening, sickle cell disease, marital decisions, sickle cell disease prevention, Nigerian government workers

## Abstract

**Background:**

In Nigeria, as in the rest of equatorial Africa, sickle cell disease (SCD) has its highest incidence and continues to cause high morbidity and early death. The condition is a major public health problem among the black race. The aim of this survey is to determine the level of knowledge about SCD and the factors associated with its prevention among local government workers in Ile-Ife.

**Method:**

This is a cross-sectional descriptive study of the knowledge about SCD, attitude towards premarital sickle cell screening and marital decisions among local government workers in Ile-Ife, Nigeria, using a self-administered questionnaire.

**Results:**

69% of study subjects had poor knowledge of SCD, while attitude towards premarital screening was favourable in 95% of the study subjects. Knowledge and attitude were significantly better among subjects with tertiary education. There was a strong positive association between attitude towards sickle cell screening and a history of undergoing screening or partner screening. Most (86.7%) of the respondents and 74.0% of their partners have had sickle cell screening. One-quarter of married and engaged respondents did not know their partner's sickle cell status. One-third to two-thirds of study subjects will continue the relationship with their partner when either or both have haemoglobinopathy.

**Conclusion:**

This study showed poor knowledge of SCD among the studied subjects. There is a need for more emphasis on health education through programmes promoting sickle cell education. In addition, the development of multifaceted patient and public health education programmes, the intensification of screening for the control of SCD by heterozygote detection, particularly during routine preplacement and premarital medical examinations, and the provision of genetic counselling to all SCD patients and carriers are vital to the identification and care of the couples at risk. These will enhance the capacity of the intending couples to make informed decisions and be aware of the consequences of such decisions. Policies are needed to ensure easily accessible community-wide sickle cell screening and premarital and genetic counselling to achieve the desired decline in new births of children with SCD.

## INTRODUCTION

Non-communicable diseases, especially genetic diseases such as sickle cell disease (SCD), are a major cause of morbidity and mortality. The sickle cell gene is known to be widespread, reaching its highest incidence in equatorial Africa, with the proportion of carriers in the global human population increasing as a result of a relatively high birth rate in the affected populations.

SCD is one of the most common single-gene disorders. About 25% of adults in Nigeria have the sickle cell gene, while the HbC trait is largely confined to the Yoruba people of south-western Nigeria, in whom it occurs in about 6%.^1^, ^2^ The prevalence of sickle cell anaemia is about 20 per 1 000 births. This means that in Nigeria alone, about 150 000 children are born annually with sickle cell anaemia. Despite recent advances in the management of SCD through improved care, re-induction of foetal haemoglobin synthesis and bone marrow transplantation, the condition continues to cause high morbidity and early death in Africa.

The chronic nature of SCD requiring life-long medical attention, expensive supportive symptomatic therapy, its specialised care, the associated high morbidity, reduction in life expectancy of the affected, poor school attendance, the potential risk of the development of drug addiction, especially to opiates, and its burden on the affected families all indicate that the condition is a major public health problem where ever its risk prevalence is high.^3^

Methods of preventing new haemoglobinopathy births include premarital screening and genetic counselling, prenatal diagnosis, preconceptional diagnosis and implantation of normal embryos after in vitro fertilisation, and in utero therapy using stem cell transplantation.^3^ Prevention of the disease through carrier identification and genetic counselling remains the only realistic approach to reduce the impact of the disease and allows better use of available resources in the low-income countries where the condition is most prevalent. Programmes of population screening and genetic counselling can have a major impact on the birth rate of children with SCD and other genetic diseases.^4^, ^5^, ^6^ The prospective control of SCD by heterozygote detection through premarital screening, which is vital to the identification of the couples at risk, is of utmost importance. The success of such a programme to a large extent depends on the SCD knowledge in the community, the understanding of the full consequences of having a sickler child, and people's attitude towards genetic screening and counselling.^7^, ^8^

In Nigeria, the local government is the tier of government closest to the people. It is responsible for primary health care, and houses the community marriage registry where notice for all marriages under the law must be filed. Its workers have considerable impact on local community beliefs, values and practices. Therefore, they constitute a key agent of change in their local communities and an important group of people to target for the introduction of community-wide interventions such as sickle cell education, screening and counselling. This study therefore set out to assess the knowledge about SCD, attitudes towards premarital sickle cell screening and marital decisions among the local government workers in Ile-Ife.

## AIM

The aim of this survey was to determine the level of knowledge about SCD and the factors associated with its prevention among local government workers in Ile-Ife.

## METHOD

The study is a cross-sectional descriptive study of all members of staff present during the study period in two randomly selected local government areas (LGAs) in Ile-Ife. A total of 320 pre-tested self-administered, structured questionnaires were handed out and 300 were returned. The response rate was 91%. The knowledge and attitude responses were assigned weighted scores. Knowledge scores of at least 50% of the maximum were classified as good. Similar attitudinal scores were classified as favourable. Lower scores were classified as poor or unfavourable respectively.

The data were analysed with the use of the Statistical Package for Social Sciences (SPSS) for Windows, Version 11.0. The results are presented using simple frequencies, cross-tabulations and charts. The level of statistical significance was taken as p < 0.05.

## RESULTS

**FIGURE 1:**
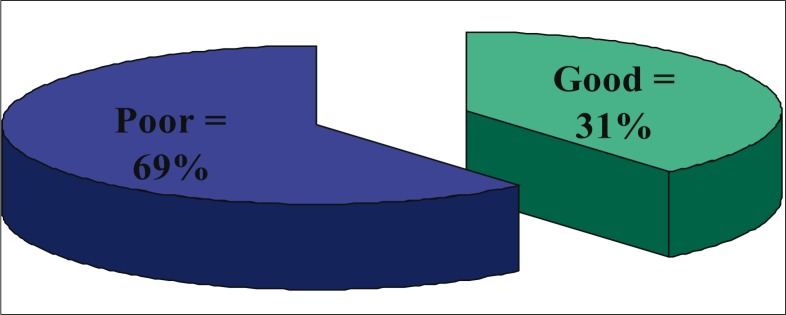
Distribution of knowedge abuout sickle cell disease in the population

**FIGURE 2:**
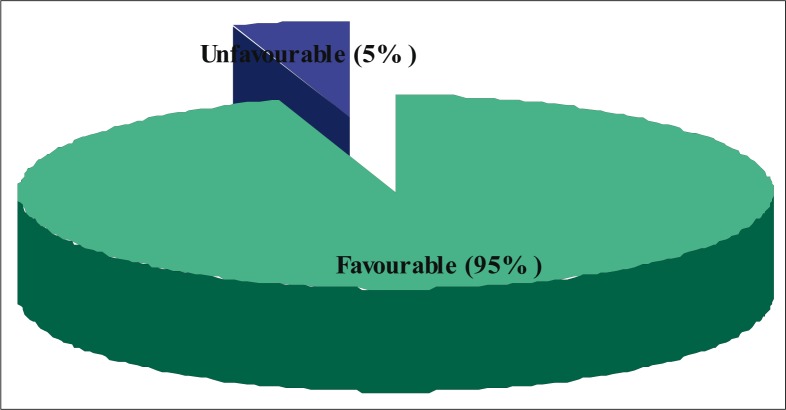
Distributition of atittude towards premarital sickle sell screening in the study population

Table 1 shows that most of the local government workers were aged 21 to 30 years (65.7%), female (51%), married (61%), had tertiary education (69.7%) and engaged in non-health-related occupations (87.3%). Figure 1 shows that the majority (69%) of the study subjects had poor knowledge of SCD, while Figure 2 shows that attitude towards premarital screening was favourable among most (95%) study subjects.

**TABLE 1:** Demographic distribution of respondents

DEMOGRAPHIC CHARACTERISTICS		n	%
**Age group**	21-30	197	65.7
	31-40	68	22.7
	41-50	27	9.0
	51-60	8	2.6
	**Total**	**300**	**100.0**
**Sex**	Male	129	43.0
	Female	171	57.0
	**Total**	**300**	**100.0**
**Occupation**	Non-health workers	262	87.3
	Health workers	38	12.7
	**Total**	**300**	**100.0**
**Level of education**	Non-tertiary	91	30.3
	Tertiary	209	69.7
	**Total**	**300**	**100.0**
**Marital status**	Single^+^	117	39.0
	Ever married	183	61.0
	**Total**	**300**	**100.0**

+ 88 (75.2%) of single respondents were engaged to a partner

Both knowledge and attitude were significantly better among subjects with tertiary education, as shown in Tables 2 to 5 (p < 0.05). Respondents’ knowledge was not associated with attitude towards premarital screening. Knowledge was also not associated with other behaviours in relation to SCD prevention. About 70% of the subjects with favourable attitude compared to 33.3% of those with unfavourable attitude were aware of their partner's sickle cell status (p = 0.007), while 59.3% of those with favourable attitude discussed their sickle cell status with their partners compared to 6.7% of those with unfavourable attitude (p = 0.001), as shown in Table 6. There was therefore a strong positive association between attitude towards sickle cell screening and a history of undergoing screening or partner screening.

**TABLE 2:** Distribution of respondents’ knowledge of sickle cell disease by socio-demographic factors

	GOOD		POOR		TOTAL				KNOWLEDGE COMMENT
					
	n	%	n	%	n	%	X^2*^	p	
**AGE (YEARS)**									
21-40	106	40.0	159	60.0	265	88.2			
41-60	11	31.4	24	68.6	35	11.7	0.955	0.329	NS
Total	117	39.0	183	61.0	300	100.0			
**SEX**									
Male	56	43.4	73	56.6	129	43.0			
Female	61	35.7	110	64.3	171	57.0	1.851	0.174	NS
Total	117	39.0	183	61.0	300	100.0			
**EDUCATION**									
Below tertiary	24	26.4	67	73.6	91	30.3			
Tertiary	93	44.5	116	55.5	204	69.7	8.753	0.003	Sig.
Total	117	39.0	183	61.0	300	100.0			
**MARITAL STATUS**									
Single	54	46.2	63	53.8	117	39.0			
Ever married	63	34.4	120	65.6	183	61.0	4.126	0.042	Sig.
Total	117	39.0	183	61.0	300	100.0			
**OCCUPATION**									
Non-health-related	95	36.3	167	63.7	262	87.3			
Health-related	22	57.9	16	42.1	38	12.2	6.530	0.011	Sig.
Total	117	39.0	183	61.0	300	100.0			

* DF = 1 NS = Not statistically significant, Sig = statistically significant

**TABLE 3:** Respondents’ mean scores of knowledge of sickle cell disease by socio-demographic factors

	KNOWLEDGE SCORE						

	n	MEAN	SD	t	p	COMMENT	
**AGE (YEARS)**						
21-40	265	6.02	3.2151			
41-60	35	5.91	3.2753	0.033	0.857	NS
**SEX**						
Male	129	6.16	3.3161			
Female	171	5.89	3.1445	0.532	0.466	NS
**EDUCATION**						
Below tertiary	91	4.51	3.5446			
Tertiary	209	6.66	3.8326	31.332	0.001	Sig.
**MARITAL STATUS**						
Single	117	6.43	3.3894			
Ever married	183	5.74	3.0808	3.306	0.070	NS
**OCCUPATION**						
Non-health-related	262	5.75	3.1942			
Health-related	38	7.76	2.8326	13.516	0.001	Sig.

NS = Not statistically significant, Sig = statistically significant

**TABLE 4:** Distribution of respondents’ attitude towards premarital sickle cell screening by socio-demographic factors

	ATTITUDE								

	FAVOURABLE		NOT FAVOURABLE		TOTAL		X^2^	p	COMMENT
					
	n	%	n	%	n	%			
**AGE (YEARS)**									
21-40	252	95.1	13	4.9	265	88.2			
41-60	33	94.1	2	5.7	35	11.7			
Total	285	95.0	15	5.0	300	100.0	.0430	0.69^+^	NS
**SEX**									
Male	119	92.2	10	7.8	129	43.0			
Female	166	97.1	5	2.9	171	57.0			
Total	285	95.0	15	5.0	300	100.0	3.608	0.57	NS
**EDUCATION**									
Below tertiary	81	89.0	10	11.0	91	30.3			
Tertiary	264	97.6	5	2.4	204	69.7			
Total	285	95.0	15	5.0	300	100.0	9.864	0.003^+^	Sig.
**MARITAL STATUS**									
Single	110	94.0	7	6.0	117	39.0			
Ever married	175	95.6	8	4.4	183	61.0			
Total	285	95.0	15	5.0	300	100.0	0.390	0.532	NS
**OCCUPATION**									
Non-health-related	247	94.3	15	5.7	262	87.3			
Health-related	38	100.0	-	-	38	12.2			
Total	285	95.0	15	5.0	300	100.0	2.290	0.230^+^	NS

* DF = 1

+ Fisher's Exact Test NS = Not statistically significant, Sig = statistically significant

**TABLE 5:** Respondents’ mean scores of attitude towards premarital sickle cell screening by socio-demographic factors

	ATTITUDE SCORE					

	n	MEAN	SD	t	P	COMMENT
**AGE (YEARS)**						
21-40	265	7.03	1.5086			
41-60	35	7.09	1.5033	0.048	0.827	NS
**SEX**						
Male	129	6.83	1.7771			
Female	171	7.19	1.2415	4.207	0.0411	Sig.
**EDUCATION**						
Below tertiary	91	6.34	1.8572			
Tertiary	209	7.34	1.2061	30.467	0.001	Sig.
**MARITAL STATUS**						
Single	117	6.97	1.6965			
Ever married	183	7.08	1.3687	0.386	0.535	NS
**OCCUPATION**						
Non-health-related	262	6.99	1.5598			
Health-related	38	7.34	0.9939	1.841	0.176	NS

NS = Not statistically significant, Sig = statistically significant

**TABLE 6:** Attitude towards premarital sickle cell screening, awareness and discussion of sickle cell status with partner

ATTITUDE TOWARDS PREMARITAL SICKLE CELL SCREENING			AWARENESS OF PARTNER'S SICKLE CELL STATUS				DISCUSSION OF SICKLE CELL STATUS WITH PARTNER			
			
			YES		NO		YES		NO	
					
	N		n	%	n	%	n	%	n	%
Favourable	285	95.0	201	70.5	84	29.5	169	59.3	116	40.7
Unfavourable	15	5.0	5	33.3	10	66.7	1	6.7	14	93.3
**TOTAL**	300	100.0	206	68.7	94	31.3	170	56.7	130	43.3
DF = 1	X^2^		9.162				16.075			
	p		0.002				0.001			

**FIGURE 3:**
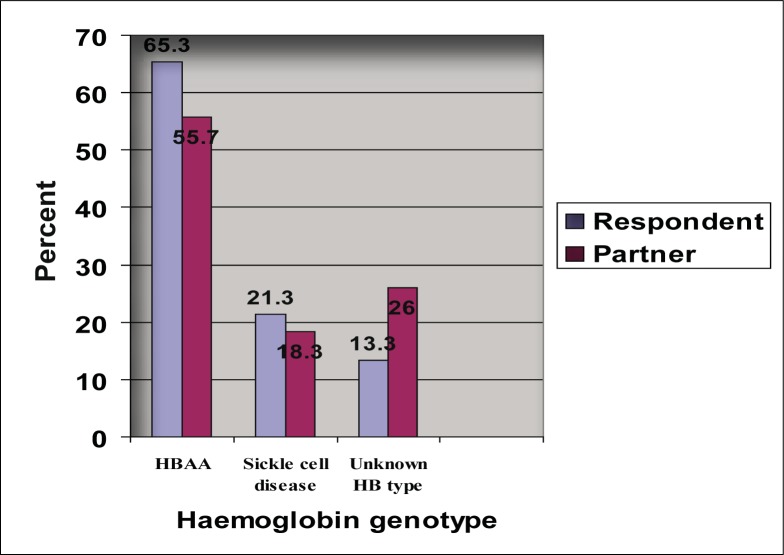
Distribution of haemoglobin genotype of respondents and their partners

**FIGURE 4:**
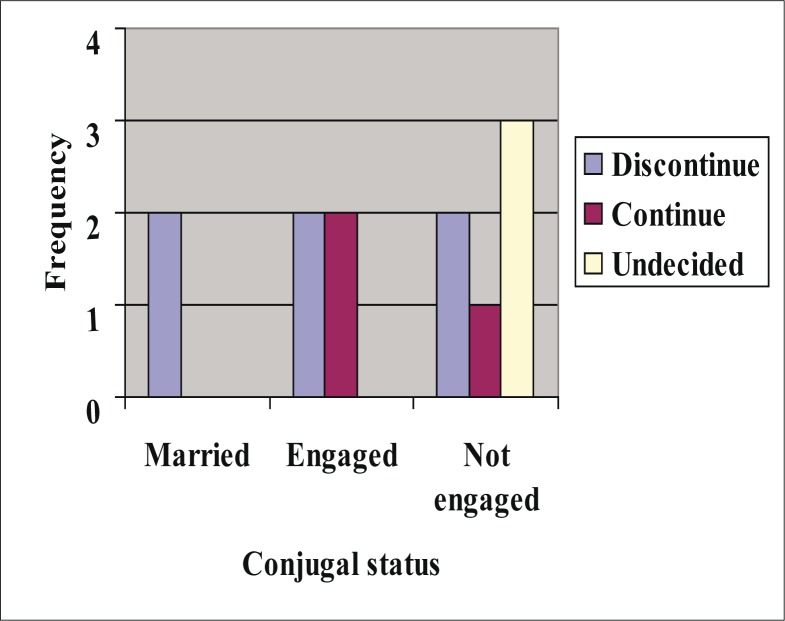
Distribution of decisions about relationship when both respondent and partner have haemoglobinopathy according to conjugal status

40 (13.3%) study subjects did not know their sickle cell status. Figure 3 shows that about 20% of respondents were aware that they have haemoglobinopathy. It also shows that an approximately similar proportion of respondents knew their partners had haemoglobinopathy. Of the 271 (90.3%) respondents who were either married or have already decided on who to marry, 202 (74.5%) knew their partner's sickle cell status. 46 (25.1%) of the 183 married respondents did not know their spouse's sickle cell status while 23 (26.1%) of the 88 respondents engaged to a marital partner (committed to a marital relationship) did not know their future partner's sickle cell status. Among those who knew both their sickle cell status and that of their partner (Table 7), high proportions (34.4–64.5%) of subjects with haemoglobinopathy stated that they will continue with conjugal relationships. Even when both partners have haemoglobinopathy, as many as 50% did not decide to discontinue the relationship. Figure 4 shows the distribution of the decisions of both partners with haemoglobinopathy according to conjugal status. Table 7 also shows that variable proportions (20–60%) took the decision to screen the other partner when one had haemoglobinopathy. Of 382 offspring of respondents whose sickle cell status was reported, 52 (13.6%) in 40 families had haemoglobinopathy (50 HBAS; 1 HBSS and 1 HBSC respectively).

**TABLE 7:** Distribution of respondents’ marital decisions in response to haemoglobinopathy

	DISCONTINUED RELATIONSHIP		CONTINUED RELATIONSHIP		SCREEN OTHER PARTNER		TOTAL	
			
	n	%	n	%	n	%	n	%
Haemoglobinopathy in respondent	6	9.4	22	34.4	36	56.3	64	100.0
HaemoglobinoPathy in Partner	5	16.1	20	64.5	6	19.4	31	100.0
	**DISCONTINUED RELATIONSHIP**		**CONTINUED RELATIONSHIP**		**UNDECIDED**		**TOTAL**	
			
	**n**	**%**	**n**	**%**	**n**	**%**	**n**	**%**
Haemoglobinopathy in both	6	50.0	3	25.0	3	25.0	12	100.0

## DISCUSSION

In this study, the majority of the 300 respondents (86.7%) and their partners (74.0%) have had sickle cell screening, as shown in Figure 3. This is not unexpected from the finding of a favourable attitude towards screening in 95% of the respondents. There is a strong positive association between attitude towards sickle cell screening and a history of undergoing screening (p < 0.05). The majority (65.7%) of subjects were 30 years of age or younger, more than half of whom were yet to marry. Even though the majority (69.7%) had tertiary education, only a small proportion of subjects (31.0%) had good knowledge of SCD. About a quarter of the married respondents and those engaged to a partner did not know their partner's sickle cell status. These findings indicate the necessity of functional education, early life sickle cell education, screening and counselling. Population screening and genetic counselling have been very successful in places like Cyprus, with almost no new births of affected children.^9^ Similarly, the affected child birth rate has fallen to about 20% of the expectation in the whole of mainland Italy and Greece.^10^ In Bahrain the establishment of genetic clinics, premarital counselling, screening of all pregnant women, newborn testing, student screening and a multifaceted population education programme have resulted in an increase in premarital counselling attendance, premarital mandatory counselling (PMC), PMC law and a reduction in the incidence of SCD from 2.1% to 0.9% – a 60% decline in incidence rate over two decades.^4^ Similarly, in Cyprus the incidence of b-thalassaemia was reduced through health education, carrier screening, premarital counselling and prenatal diagnosis. This success has been reproduced in the control of other genetic diseases using carrier screening and premarital counselling, either voluntarily or by legal enforcement.^5^, ^6^

One-third to two-thirds of the subjects in this study will continue the relationship with their partner when either or both have haemoglobinopathy, emphasising the urgent need for focused health education as the foundation for genetic counselling before marriage. As expected, both knowledge and attitude were significantly better in the group with tertiary education compared to the group with less education. A favourable disposition towards screening was associated with awareness of the partner's sickle cell status and discussion of haemoglobin sickle cell status with the partner (p < 0.05). With a generally favourable attitude towards sickle cell screening and a significant positive influence of attitude on screening, partner discussion and marital decisions, the universal provision of and emphasis on sickle cell screening and genetic counseling especially long before marriage as recommended by WHO^11^ will facilitate informed rational marital decisions and reproductive behaviour that will lead to a sharp decline in new births of children with SCD.^12^

### Conclusion

This study showed poor knowledge of SCD among the studied subjects. There is a need for more emphasis on health education through programmes promoting sickle cell education using the mass media, the school health services and by including the subject in the physical and health education curriculum of primary and secondary schools in the country. In addition, the development of multifaceted patient and public health education programmes, the intensification of screening for the control of SCD by heterozygote detection, particularly during routine preplacement and premarital medical examinations, and the provision of genetic counselling to all sickle cell patients and carriers are vital to the identification and care of the couples at risk. These will enhance the capacity of the intending couples to make informed decisions, to improve their communication about their sickle cell status and to be aware of the risks of having children with SCD and of the consequences of such decisions. Of particular importance are carefully monitored policies to ensure easily accessible community-wide sickle cell screening and premarital and genetic counselling to achieve the desired decline in new births of children with SCD.
